# Emotional Support and Subjective Cognitive Decline: The Moderating Role of Adverse Childhood Experiences

**DOI:** 10.1093/geroni/igaf122.4108

**Published:** 2025-12-31

**Authors:** Boram Lee, Jeehoon Kim

**Affiliations:** Ewha Womans University, Seoul, Republic of Korea; Idaho State University, Pocatello, Idaho, United States

## Abstract

Emotional support is well-known to protect against older adults’ cognitive decline; however, it remains unclear whether this protective effect differs depending on exposure to adverse childhood experiences (ACEs), particularly given the unanticipated stress during the COVID-19 pandemic. We examined the associations between emotional support, ACEs, and subjective cognitive decline (SCD) in American older adults. We analyzed the 2022-2023 Behavioral Risk Factor Surveillance System data for White, Black, and Hispanic adults aged 65 + (n = 53,261). Logistic regressions assessed associations among emotional support, ACEs and SCD, including an interaction of emotional support and ACEs, after adjusting for sociodemographic characteristics, depression, self-reported health status, health insurance, and routine medical care utilization. Overall, 15.3% of the sample reported SCD in the past 12 months. In adjusted models, high levels of emotional support were associated with lower odds of SCD (Adjusted OR = 0.51, 95% CI = 0.45-0.59), whereas ACE exposure was associated with higher odds of SCD (Adjusted OR = 1.27, 95% CI = 1.13-1.45). The protective association of emotional support was stronger among older adults without ACEs than among those with a history of ACEs (Adjusted OR = 1.39, 95% CI = 1.21-1.59). These findings suggest that emotional support provides a buffer against SCD in later life, but this protective effect is diminished for older adults with a history of ACEs. The reduced emotional support availability among ACE-exposed individuals may reflect smaller social networks and compromised social relationship quality established earlier in life. This underscores the need for targeted interventions to address both cognitive and social deficits in older adults exposed to ACEs.

